# Narcotic Nitrogen Effects Persist after a Simulated Deep Dive

**DOI:** 10.3390/medicina60071083

**Published:** 2024-07-02

**Authors:** Sven Dreyer, Johannes Schneppendahl, Martin Hoffmanns, Thomas Muth, Jochen D. Schipke

**Affiliations:** 1 Hyperbaric Oxygen Therapy (HBO), University Hospital Düsseldorf, 40225 Düsseldorf, Germany; sven.dreyer@med.uni-duesseldorf.de; 2 Klinik für Orthopädie und Unfallchirurgie Evangelisches Krankenhaus, 45468 Mülheim/Ruhr, Germany; johannes.schneppendahl@evkmh.de; 3 Klinik für Handchirurgie und Unfallchirurgie, University Hospital Düsseldorf, 40225 Düsseldorf, Germany; martin.hoffmanns@med.uni-duesseldorf.de; 4 Institute of Occupational, Social and Environmental Medicine, Centre for Health and Society, Faculty of Medicine, Heinrich-Heine-Universität Düsseldorf, 40225 Düsseldorf, Germany; thomas.muth@uni-duesseldorf.de; 5 Research Group Experimental Surgery, University Hospital Düsseldorf, Moorenstrasse 5, 40225 Düsseldorf, Germany

**Keywords:** hyperbaric chamber, nitrogen narcosis, Romberg test, fine motor skills

## Abstract

*Background and Objectives*: Scuba divers often experience persistent inert gas narcosis (IGN) even after surfacing. This study aimed to test the hypothesis that breathing oxygen (O_2_) before surfacing can reduce postdive IGN. *Materials and Methods*: A group of 58 experienced divers underwent a 5 min dive at a depth of 50 m in a multi-place hyperbaric chamber. They were decompressed using air (air group). Another group of 28 divers (O_2_ group) breathed 100% O_2_ during the end of decompression. Prior to and after the dive, all participants performed the Sharpened Romberg test (SRT) and a modified tweezers test. *Results*: In the air group, the number of positive SRT results increased postdive (47% vs. 67%), indicating a greater impairment in the vestibular system (Cohen’s d = 0.41). In the O_2_ group, the percentage of positive SRT results remained constant at 68% both before and after the dive. In terms of the modified tweezers test, the air group showed no significant change in the number of picked beads (40 ± 9 vs. 39 ± 7), while the O_2_ group demonstrated an increase (36 ± 7 vs. 44 ± 10) (Cohen’s d = 0.34). *Conclusion*: The results reveal that the SRT revealed a negative effect of nitrogen (N_2_) on the vestibular system in the air group. The increased number of beads picked in the O_2_ group can be attributed to the learning effect, which was hindered in the air group. Consistent with our hypothesis, breathing O_2_ during decompression appears to reduce postdive IGN.

## 1. Introduction

Albert Behnke described the narcotic effects of nitrogen as early as 1935 [1]. However, the first description of this effect was made about 90 years earlier by the French physician Colladon, who reported that a descent in a diving bell resulted in his feeling ‘…as though I had drunk some alcoholic liquor’ [2].

Initially, the prevailing theory proposed that narcosis occurs when the volume of a hydrophobi site expands beyond a critical point due to the absorption of N_2_ molecules. More recently, the protein theory has gained recognition, suggesting a direct interaction between N_2_ and neurotransmitter receptors [3,4].

There is a commonly held belief that N_2_ narcosis is similar to alcohol intoxication [5,6]. If this is the case, narcotic effects should gradually manifest with increasing partial N_2_ pressure and slowly diminish as N_2_ molecules are eliminated from the tissues. The elimination process is prolonged, and N_2_ bubbles can be detected for up to two hours [7,8,9], or even longer [10], with the peak bubble formation occurring approximately 40 to 50 min after surfacing [11]. Therefore, as long as bubbles are present, N_2_ has not been completely eliminated. Similarly, the effect of an anaesthetic does not end abruptly.

Scuba divers who breathe air encounter these intoxicating depths, experiencing a severe form of N_2_ narcosis when diving beyond 40 m, which becomes more pronounced with increasing depths. It is often overlooked that narcotic N_2_ symptoms do not suddenly manifest at a specific depth, and often a dive buddy will notice changes in the affected diver before they become aware themselves [12].

These symptoms progressively worsen from depths exceeding 10 m, leading to a reduction in cognitive and fine motor skills. Importantly, the narcotic effects of N_2_ do not disappear immediately after surfacing [3,13], potentially impairing rapid reactions and decision making when required.

The Romberg test (RT) is employed to assess the healthy functioning of the dorsal columns of the spinal cord [14]. Apart from balance testing, it is also used as an indicator of possible impairment due to alcohol consumption [15] or drug use while driving [16,17]. Furthermore, the RT is valuable for documenting confirmed or suspected cases of neurological decompression sickness [18,19], making it suitable for use in hyperbaric chambers or diving scenarios with increased ambient pressures. Given these factors, we utilized this simple test to detect possible N_2_-induced postdive narcosis.

Manual dexterity tests are commonly used in occupational therapy to diagnose fine motor disorders in injured patients and evaluate the adaptability of healthy individuals in terms of fine motor skills [20], such as those used in dentistry [20] and surgery [21]. Increasing partial N_2_ pressures (pN_2_) have been shown to impair manual dexterity [22]. Considering the potential detrimental impact of N_2_ narcosis on divers’ manual dexterity, it was concluded that the test of manual dexterity is sensitive to the effects of depth [23]. The O’Connor tweezer dexterity test is a standardized, timed test that assesses finger and eye–hand coordination by requiring the placement of pins in holes using tweezers [24].

The aim of this study is to examine the effects of decompressing with oxygen during the last 30 min following a simulated 5 min dive to a depth of 50 m, in comparison to decompressing with air.

The hypothesis to be tested was that breathing oxygen would reduce postdive N_2_-induced narcotic effects.

## 2. Methods

A total of 86 male professional firefighters from different cities participated in this study, with an average age of 36.4 ± 6.7 years. Their average body height was 184 ± 8 cm, and they had a body mass of 88 ± 11 kg, resulting in a BMI of 26.6 ± 3.5 kg/m², indicating a normal weight considering the high fitness level required for their demanding activities. No significant differences were found in their demographic characteristics, except for age (Table 1). The study was approved by the Ethical Committee of the Medical Faculty of the Heinrich Heine University Düsseldorf (Study No. 2020-998), 18 August 2020.

**Figure 1:**
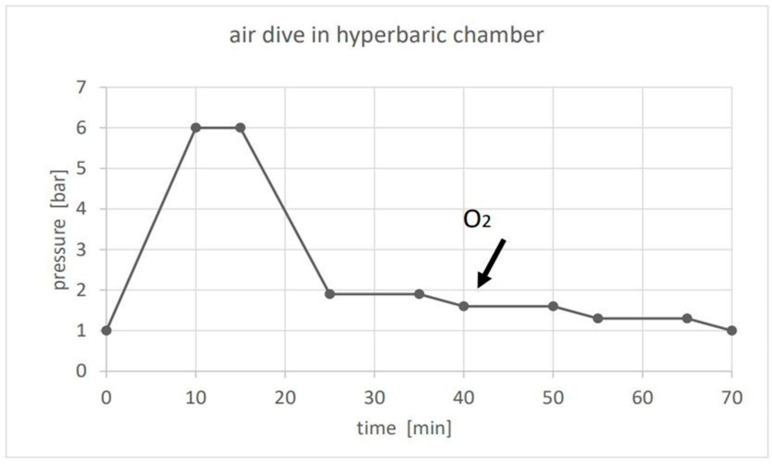
The air dive in a hyperbaric chamber lasted 70 min. During decompression, one of the two groups started breathing 100% O_2_ at 1.6 bar (see arrow). That pressure was decreased to 1.3 bar and further to 1.0 bar at the end of the dive.

All participants simulated a dive by undergoing a 5 min stay at a depth of 50 m within a 12-person hyperbaric chamber (Figure 1). Of the participants, 58 underwent decompression using air (air group), while 28 underwent decompression using 100% oxygen via breathing masks (O_2_ group) during the last 30 min of decompression, following a specific protocol: 10 min at 1.6 bar, 5 min to 1.3 bar, 10 min at 1.3 bar, and 5 min to 1 bar.

It is important to note that all participants engaged in the simulated dive as part of their ongoing training as firefighter divers, and not specifically for this study.

Prior to the hyperbaric session, all 86 participants possessed the necessary permissions to work in compressed air, in accordance with the relevant regulations [25].

Before and after the simulated dives, all participants underwent a neurological examination, which included four tests. Firstly, the finger-to-nose test was conducted to evaluate smooth and coordinated upper-extremity movement, requiring participants to touch their nose with their index finger [26]. This simple test was mainly conducted to familiarize the participants with the similar procedure used in the subsequent Romberg tests. Secondly, the Romberg test was performed to assess participants’ actual balance [27]. For this test, participants stood upright with shoes on, arms stretched forward, palms facing upwards, and feet positioned parallel to each other. When instructed, they closed their eyes.

Furthermore, the Sharpened Romberg test (SRT), also known as the tandem Romberg Test, was administered, which was similar to the traditional Romberg test except for the fact that participants stood heel-to-toe [28,29,30]. Both tests were conducted once and lasted for 15 s, with participants performing them first with eyes open and then with eyes closed. To prevent participants from falling, an observer stood behind each participant during the tests. The entire test series was recorded on video and later analyzed by a blinded investigator, who determined whether the Romberg test results were “negative” or “positive” based on any motion, irregular swaying, or toppling over.

Additionally, all participants completed a modified tweezers test to evaluate their fine motor skills [31,32]. Using their dominant hand, participants picked up one plastic bead (∅: 4 mm) at a time from one shell and placed them into another shell within 60 s. The beads had a smooth surface.

The postdive tests were carried out immediately after the participants had exited the hyperbaric chamber.

### Statistics

The data were assessed for normal distribution using the Kolmogorov–Smirnov test. If the data were normally distributed, they are presented as mean ± standard deviation (SD); otherwise, they are presented as median and range. Significant differences were determined using a *p*-value threshold of <0.05.

Differences in demographic data between the two groups and differences observed prior to the dive in relation to the Sharpened Romberg test (SRT) and the modified tweezers test were assessed using the non-parametric Mann–Whitney *U* test (SPSS Statistics 24, IBM, New York, NY, USA).

For the main outcomes, which include the N_2_-induced effects on postural stability and fine motor skills, no significance levels or *p*-values are reported to evaluate treatment-induced differences. 

In addition, effect sizes were calculated using Cohen’s d [33] and classified as follows: large effect |d| = 0.8, moderate effect |d| = 0.5, and small effect |d| = 0.2. The calculations were performed using SPSS Statistics 24 (IBM, New York, NY, USA).

## 3. Results

(1) The finger-to-nose test—left and right arm—was successfully completed by almost all participants, indicating that there were no significant differences between the two groups of young members of the diving team from the Municipal Fire Brigade, both before and after the dives.

(2) The original Romberg test yielded somewhat inconsistent results, as some participants in both groups tested positive before the dive and negative after the dive.

(3) The Sharpened Romberg test (SRT) consistently showed a higher number of positive participants compared to the regular Romberg test. In the air group, the percentage of positive SRT results increased significantly from 47% before the dive to 67% after the dive (Cohen’s d = 0.41). In the O_2_ group, the percentage of positive SRT results remained at 68% both before and after the dive. Prior to the dive, the percentage of positive participants in the O_2_ group was significantly higher than in the air group (*p* < 0.05).

In terms of the number of transferred beads, the air group had an average of 42 ± 10 before the dive and 41 ± 8 (n.s.) after the dive. In contrast, the O_2_ group had an average of 36 ± 7 before the dive and 42 ± 9 after the dive (Cohen’s d = 0.74). Prior to the dive, the number of beads in the air group was significantly greater than in the O_2_ group (*p* < 0.05).

## 4. Discussion

The present study yielded two main findings: administering oxygen during the last 30 min of decompression following a 5 min stay at a depth of 50 m resulted in improved results in the Sharpened Romberg test (fewer negative outcomes) and preserved a learning effect in the modified tweezers test (bead picking). These results support the concept of late postdive impairment [34] and suggest that oxygen administration during decompression facilitates the washout of nitrogen according to Henry’s law.

### 4.1. Study Limitations

While the comparison between inert gas narcosis (IGN) and alcohol intoxication may be tempting [5,6], it is important to recognize that N_2_ washin and washout is a physical process—similar to an anesthetic—whereas alcohol is metabolized by the liver. However, both processes exhibit similar time courses, with neither starting nor ending abruptly. We therefore hold residual nitrogen, similar to residual alcohol, responsible for the restricted performance.

It is worth noting that centering our focus on nitrogen as the cause of any postdive changes in our tests may not be entirely accurate. Admittedly, persistent vestibular stimulation due to repeated ear clearing maneuvers during compression and venting during decompression might have influenced the outcomes of the Sharpened Romberg test (RST). However, these maneuvers would have affected both groups equally.

Additionally, it would be valuable to include a third group breathing a gas mixture of 21% oxygen and 79% helium to provide a more comprehensive understanding. Such a study could be conducted in the future to draw even more definitive conclusions regarding whether the changes observed in the air group were specifically due to postdive N_2_-related narcosis.

Another aspect to consider is the age difference between the air and O_2_ groups, with the latter being, on average, eight years older. Performance in the Romberg test has been reported to decline over decades [35]. A recent study confirms this statement but shows that there are almost no differences in the Romberg test among 30 year olds [36]. Therefore, we believe that age ultimately did not contribute to the relatively high number of positive outcomes in the SRT. On the other hand, fine motor skills also decline over the course of life. In a study on age dependence in juggling, a difference of about 12% was observed between those in their early 30s and those in their late 30s. Therefore, part of the difference in the collected beads observed here (~16%) could be attributed to the different ages.

A final aspect concerns the modified tweezer test. It might be that, within the given time of 60 s, no more than a maximum of 40 beads can be transferred in total from one shell to another. Then, an improvement in the air group after the dive due to a learning effect would become impossible. However, this consideration is not convincing, as some participants were able to achieve values of even up to 70 beads within the time given. 

### 4.2. Sharpened Romberg Test (SRT)

The air group exhibited a relatively high percentage of “positive” results before the simulated dive, which increased from 47% to 67% after surfacing. In contrast, the percentage of positive results in the O_2_ group remained unchanged after the dive, indicating that balance was not further impaired.

Two factors may have influenced our results. Firstly, the Romberg tests are known to have a learning effect [28,29]. We partially accounted for this effect by performing both the original Romberg test (RT) and the SRT with eyes open first, followed by eyes closed. Moreover, we only present data from the SRT, considering the original RT as a practice round. Clinically, a maximum time of 30 s has been suggested for maintaining the position with eyes closed [37]. Accordingly, young adults should be able to successfully perform this test for 30 s [36]. The latter author recently proposed scoring the test by counting the seconds the participant can stand with eyes closed, even as short as 6 s [38], indicating early movement. We report relatively high “positive” results within 15 s due to the challenging conditions of our test. Participants were in the tandem position with arms stretched forward and palms facing upwards. Additionally, we only distinguished between “no motion” and “some sort of motion.” Therefore, we suggest that the significantly increased number of “positive” results in the air group is attributable to postdive N_2_ narcosis.

### 4.3. Modified Tweezers Test

In this test, which assessed fine motor skills during the transfer of small beads, the regions of the frontal lobe responsible for such skills were engaged. We firmly believe in a learning effect being associated with this activity. Since no such effect was observed in the air group, we propose that any improvement was counteracted by persistent IGN effects. The clear increase in the number of shifted beads in the O_2_ group by 22% further supports the concept of a learning effect.

The results from both tests suggest that the postdive effects of IGN can be mitigated by breathing oxygen towards the end of the dive [39]. This beneficial effect can be attributed to simple physics, as the diffusion gradient for nitrogen increases once no more nitrogen is inspired.

### 4.4. Oxygen vs. Nitrogen Effects

Regarding the effects of oxygen versus nitrogen, oxygen has been recognized as a potent modulator of IGN symptoms [40]. Consequently, the effects of hyperoxia on cognitive performance need to be addressed. One older study showed no effect when breathing pure oxygen at the surface [41]. However, subsequent studies demonstrated that oxygen administration can selectively enhance certain aspects of cognitive performance [42], with increased brain activity and improved multitasking performance reported during hyperoxia [43]. It is important to note that these studies were conducted under normobaric conditions without prior dives and IGN effects.

Other studies investigating the effects of increased oxygen partial pressures have consistently reported beneficial effects on neurocognitive performance [34], such as long-term memory [44] and critical flicker fusion frequency [45] during the dive. In our study, no such effects developed since 100% oxygen was administered only during the last 30 min of decompression, resulting in an oxygen partial pressure of 1.6 bar at the beginning of administration and 1.0 bar at the end of decompression. Therefore, we propose that, in accordance with Behnke’s lipid theory [1], the reported beneficial effects of oxygen were due to the washout of nitrogen from lipid bilayers of cellular membranes [4,46]. Alternatively, if the protein theory [4] proves to be correct, a faster nitrogen washout would equally improve the persistent cerebral impairment described to last for 30 min postdive [3].

### 4.5. Fatigue

This phenomenon is often reported to be reduced after nitrox dives [40]. Thus, it is worth considering whether fatigue, characterized by a lack of energy and motivation, may have influenced our results. Similar to our findings of negative IGN effects lasting a maximum of 30 min postdive, fatigue also persists for 30 to 60 min after surfacing [40]. However, the effects of oxygen-enriched breathing gases on fatigue remain uncertain [35], even when breathing nitrox throughout the entire dive. Therefore, we do not propose that fatigue significantly affected our results.

## 5. Summary/Conclusions

As nitrogen cannot rapidly diffuse out of the tissues, narcotic symptoms continue to persist after surfacing. The Sharpened Romberg test revealed an adverse effect of nitrogen on the vestibular system in the air group. Additionally, the increased number of beads in the O_2_ group can be attributed to the learning effect, which was absent in the air group likely due to lingering nitrogen effects in the cerebellum. It is acknowledged that a decompression protocol incorporating oxygen stops the acceleration of the nitrogen washout from the tissues. This study confirms the postdive persistence of inert gas narcosis effects and supports our hypothesis that breathing oxygen towards the end of decompression also provides a beneficial effect also for simulated dives. Therefore, we recommend the implementation of such oxygen stops as they have the potential to reduce post-dive, nitrogen-related cognitive impairments. In situations where oxygen stops are not feasible, such as during dive safaris with repetitive diving, the accumulating restrictions could lead to slower and less accurate reactions to extreme situations after the completion of a dive.

## Figures and Tables

**Table 1:** Demographics of the firefighter divers. Data for age are mean and range; other variables: mean ± SD.

		Air-Group n = 58	O_2_-Group n = 28	*p*
age	[years]	30 (21–54)	38 (26–58)	<0.05
height	[cm]	184 ± 8	182 ± 7	n.s.
body mass	[kg]	87 ± 10.9	89 ± 11.1	n.s.
BMI	[kg/m^2^]	26.0 ± 2.6	27.5 ± 3.6	n.s.

## Data Availability

Data are available at the Institute of Occupational-, Social- and Environmental Medicine, Centre for Health and Society, Heinrich-Heine-Universität Düsseldorf.
